# A novel mechanism linking memory stem cells with innate immunity in protection against HIV-1 infection

**DOI:** 10.1038/s41598-017-01188-3

**Published:** 2017-04-21

**Authors:** Yufei Wang, Trevor Whittall, Stuart Neil, Gary Britton, Mukesh Mistry, Supachai Rerks-Ngarm, Punnee Pitisuttithum, Jaranit Kaewkungwal, Sorachai Nitayaphan, Xuesong Yu, Alicia Sato, Robert J. O’Connell, Nelson L. Michael, Merlin L. Robb, Jerome H. Kim, Thomas Lehner

**Affiliations:** 1 Mucosal Immunology Unit, Kings College, London, UK; 2 Department of virology, Kings College, London, UK; 3 Department of Disease Control, Ministry of Public Health, Nonthaburi, Thailand; 4 Faculty of Tropical Medicine, Mahidol University, Bangkok, Thailand; 5 Armed Forces Research Institute of Medical Sciences, Bangkok, Thailand; 6 Statistical Center for HIV/AIDS Research and Prevention, Fred Hutchinson Cancer Research Center Seattle, Washington, USA; 7 US Military Research Program, Walter Reed Army Institute of Research, Silver Spring, USA; 8 Henry Jackson Foundation for the Advancement of Military Medicine, Bethesda, MD USA; 9 International Vaccine Institute, Seoul, Korea

## Abstract

HIV infection affects 37 million people and about 1.7 million are infected annually. Among the phase III clinical trials only the RV144 vaccine trial elicited significant protection against HIV-1 acquisition, but the efficacy and immune memory were inadequate. To boost these vaccine functions we studied T stem cell memory (TSCM) and innate immunity. TSCM cells were identified by phenotypic markers of CD4^+^ T cells and they were further characterised into 4 subsets. These expressed the common IL-2/IL-15 receptors and another subset of APOBEC3G anti-viral restriction factors, both of which were upregulated. In contrast, CD4^+^ TSCM cells expressing CCR5 co-receptors and α4β7 mucosal homing integrins were decreased. A parallel increase in CD4^+^ T cells was recorded with IL-15 receptors, APOBEC3G and CC chemokines, the latter downmodulating CCR5 molecules. We suggest a novel mechanism of dual memory stem cells; the established sequential memory pathway, TSCM →Central →Effector memory CD4^+^ T cells and the innate pathway consisting of the 4 subsets of TSCM. Both pathways are likely to be activated by endogenous HSP70. The TSCM memory stem cell and innate immunity pathways have to be optimised to boost the efficacy and immune memory of protection against HIV-1 in the clinical trial.

## Introduction

The global human immunodeficiency virus (HIV-1) pandemic continues, and an effective vaccine has so far not been produced. A number of HIV phase III vaccine trials have been carried out but only the RV144 prime-boost trial achieved significant, though limited protection of 31.2% against HIV acquisition^1^. The vaccine induced mostly antibody binding and ADCC (antibody dependent cytotoxicity). Investigations into the immune correlates of protection showed an inverse correlation between binding IgG antibody levels to the HIV-1gp120 variable regions 1 and 2 (V1V2) and the risk of HIV-1 infection^2–4^. A surprising finding was that IgA antibodies against envelope were directly associated with lack of protection, possibly by blocking certain HIV specific IgG effector functions^5^. A comprehensive functional analysis of vaccine-induced CD4^+^ T cell responses demonstrated polyfunctional antigen-specific cellular immune responses; CD154 expression, IL-2, IL-4, IFN-γ, and TNF-α cytokines, which were inversely correlated to HIV-1 infection^4, 6, 7^. The CD4^+^ T cells directed against HIV-1 envelope^2–4^ were mostly HIV-env specific CD45RO^+^ CCR7^−^ effector memory T cells^4^.

A robust immunological memory is critical for the function of any vaccine and may have been inadequate in the RV144 vaccine. The efficacy of protection of HIV-1 acquisition decreased from 60% in the 1^st^ year, to 36% in the 2^nd^ and 32.3% in the 3^rd^ year^8^, despite expressing significant Env-specific CD4^+^ effector memory T cells^4^. This led us to examine long-term T stem cell memory (TSCM) cells, defined by expressing CD45RO^−^ CCR7^+^ CD62L^+^ CD95^+^ T cell phenotypic markers^9, 10^. TSCM cells were studied by polychromatic flow cytometry^9, 10^ and have been reported in mice, NHP (non-human primates) and humans, but this is the first investigation of the effect of vaccination on TSCM. We hypothesised that there are subsets of CD4^+^ TSCM cells associated with innate immune responses to the RV144 vaccine and we analysed these cells in relation to the central and effector memory T cells.

HIV-1 infection is inhibited by two well defined naturally occurring mechanisms. Homozygous 32-bp CCR5 deletion^11, 12^ and allo-immunity^13–16^ have been demonstrated by HIVgp140/HSP70 immunization and allo-immunization of humans and NHP, inducing CC chemokines, which downmodulate CCR5^14–16^. A third type of natural immunity has been identified in sooty mangabeys, which acts as a natural host for SIV infection, in which high concentrations of SIV persist, the CD4 cell count does not fall and the animals remain healthy without developing AIDS^17^. The key feature is a low level of cell surface expression of CCR5 in long-lived CD4^+^ T central and memory TSCM but high level of CCR5 in the effector memory cells^17^. Similar changes have been described in non-progressing HIV-1 infected people, who remain healthy despite high viral load and express low levels of HIV DNA in CD4^+^ TSCM^18^. Recently non-progressing HIV-1 infected children also seem to share the features found in SIV infected sooty mangabeys^19^. These immune mechanisms may play a significant role in early control of HIV infection by affecting the efficiency of mucosal HIV transmission and dissemination as well as influencing acute viral replication^20, 21^.

Innate immunity may be manifested by upregulation of CC chemokines, eliciting downmodulation of CCR5 co-receptors, which inhibits pre-entry HIV-1^22–25^. This is often followed by increase in innate retroviral restriction factors, such as A3G and tetherin, inhibiting post-entry HIV-1^25, 26^. A number of intracellular host-encoded HIV-1 restriction factors have been described, blocking viral fusion by interfering with viral RNA reverse transcription and post-integration restriction and adherence. Some of the most significant restriction factors of HIV replication are APOBEC 3 G (A3G) or F protein^27^, TRIM5-α^28, 29^, Tetherin^30, 31^, SAMHD1^32^ and MX2^33, 34^, which are largely stimulated by type 1 interferons (IFN). Mucosal immunization of NHP with HSP70 linked to SIV antigens may also upregulate A3G^35^ and inhibit Vif mediated ubiquitination of A3G^36^.

In this study of the RV144 HIV-1 vaccine trial, in which ALVAC-HIV and AIDSVAX B/E were used we found significant increases in CD4^+^ TSCM cells, which were further differentiated into CD122^+^, A3G, CCR5^+^ and α4β7 expressing subsets of cells. Significantly, enhanced early innate immunity may offer initial resistance to infection and also boost subsequent HIV-1-specific adaptive cellular and antibody responses^25, 26, 35, 37, 38^. We suggest a novel dual pathway of CD4^+^ memory stem cells; the established sequential memory pathway 1 TSCM →CMC →EMC, and innate pathway 2, in which TCSM generate subsets of IL-2/IL-15 receptors (CD122), A3G, CCR5 and α4β7 innate immune cells. The mechanism of this dual memory stem cells and innate immunity is likely to be activated by endogenous HSP70, which is the hallmark of cellular stress.

## Results

### The effect of vaccination on CD4^+^ and CD8^+^ T memory stem cells

**Figure 1:**
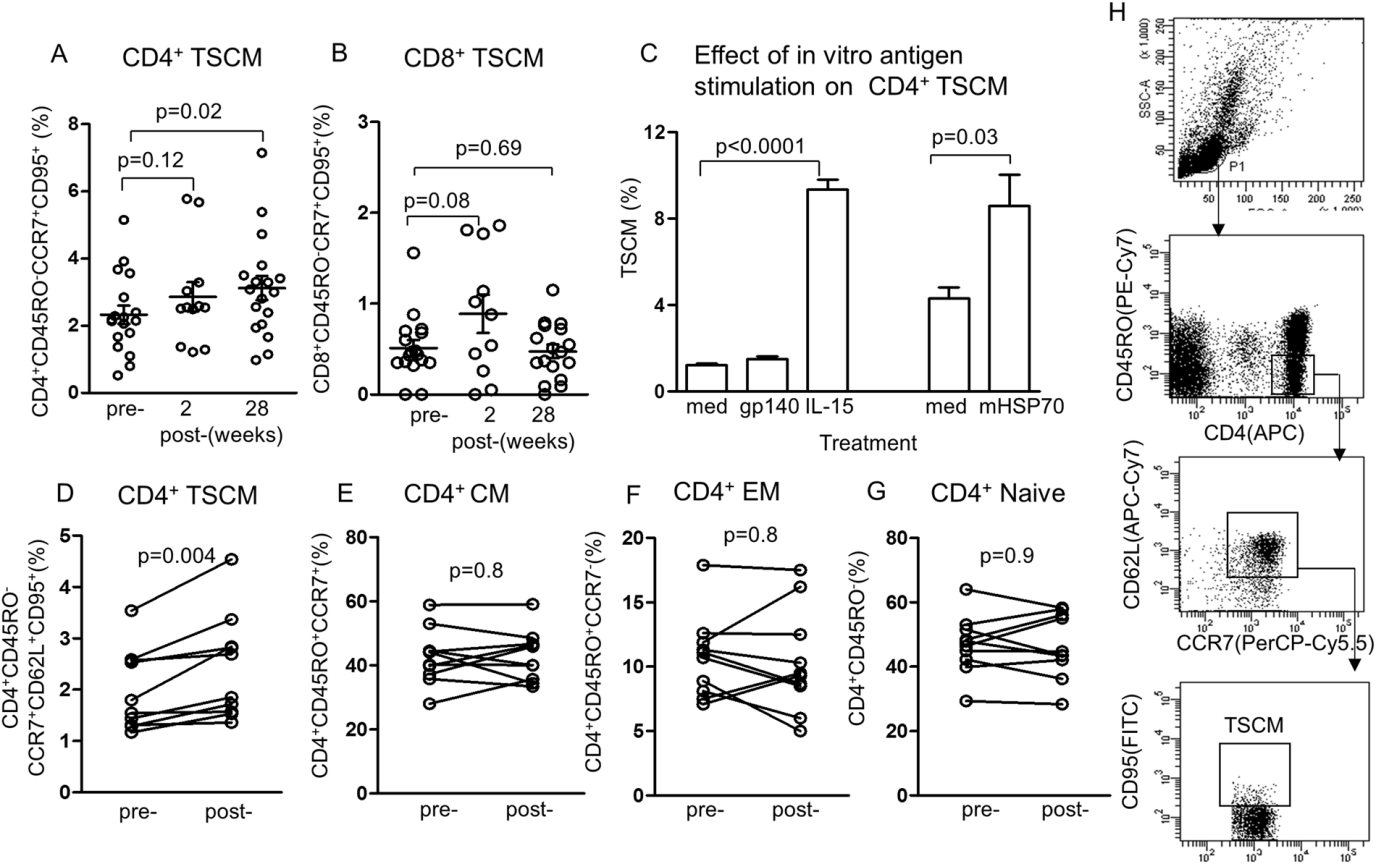
Identification of ﻿TSCM CD45RO^−^ CCR7^+^ CD95^+^ CD4^+^ and CD8^+^ T cells. (**A**) CD4^+^ TSCM and (**B**) CD8^+^ TSCM cells pre- immunization, 2 and 28 weeks after the last immunization (n = 12–18), all from different immunized subjects. (**C**) The specificity of TSCM was examined by *in vitro* stimulation with HIVgp140 and compared with recombinant IL-15 and microbial (m) HSP70. (**D**) Shows significant increase in CD4^+^ TSCM 28 weeks after the last immunization in the vaccinated cohort, but not in the CCR7^+^ central (CM), CCR7- effector memory (EM) or naïve CD4^+^ T cells (n = 10 in each group, (**E**,**F**,**G**). (**H**) Identification of TSCM and gating.

CD4^+^ and CD8^+^ T cells have been studied in the RV144 trial^4^ and demonstrated mostly CD4 CD45RO^+^ CCR7^−^ effector memory T cells^39^, responding to epitopes within the V2 region of the HIV-1 envelope. Here we have asked 2 related questions, 1) does immunization with the vaccine elicit CD4 and/or CD8 TSCM. 2) Do the TSCM express specific phenotypic markers. We have initially examined PBMC taken before immunization and 2 and 28 weeks after the last immunization. We found an increase in the population of CD4^+^ CD45RO^−^ CCR7^+^ CD95^+^ TSCM cells (2.33 ± 0.27) before vaccination to 2.86 ± 0.43 (p = 0.12) at 2 weeks and (3.12 ± 0.36 p = 0.018) at 28 weeks after the last vaccination (Fig. 1A). CD8^+^ TSCM cells were lower than those of CD4^+^ TSCM before vaccination and though they increased slightly 2 weeks after vaccination (p = 0.08), they fell to the pre-vaccination level 28 weeks after vaccination (Fig. 1B). However, the possibility will have to be considered that the vaccine might favour only the CD4^+^ and elicits limited CD8^+^ TSCM. The CD8^+^ T cells were not studied further in this paper.

To find out if the TSCM were elicited by HIVgp140 or the Canary pox/ALVAC stress component of the vaccine, cells were taken after immunization and stimulated *in vitro* with HIVgp140, which showed a negligible increase in TSCM (Fig. 1C), compared with recombinant IL-15 or microbial HSP70. This suggests that HIVgp140 was not critical in the induction of CD4^+^ TSCM, unlike IL-15 and HSP70. We repeated the CD4^+^ TSCM assays in further samples from 10 vaccinees and 7 placebo controls, pre- and 28 weeks post-immunization (Fig. 1D), This confirmed significant increase of CD4^+^ TSCM 28 weeks after the last immunization in the vaccinated cohort (p = 0.004, Fig. 1D). However, CCR7^+^ central, CCR7^−^ effector memory and naïve CD4^+^ CD45RO^−^ T cells from the same samples of blood showed no significant difference after vaccination (Fig. 1E–G). Identification of TSCM and gating are shown in Fig. 1H. Placebo controls showed no significant difference in TSCM (p = 0.34).

### The effect of vaccination on TSCM cells expressing CD122

**Figure 2:**
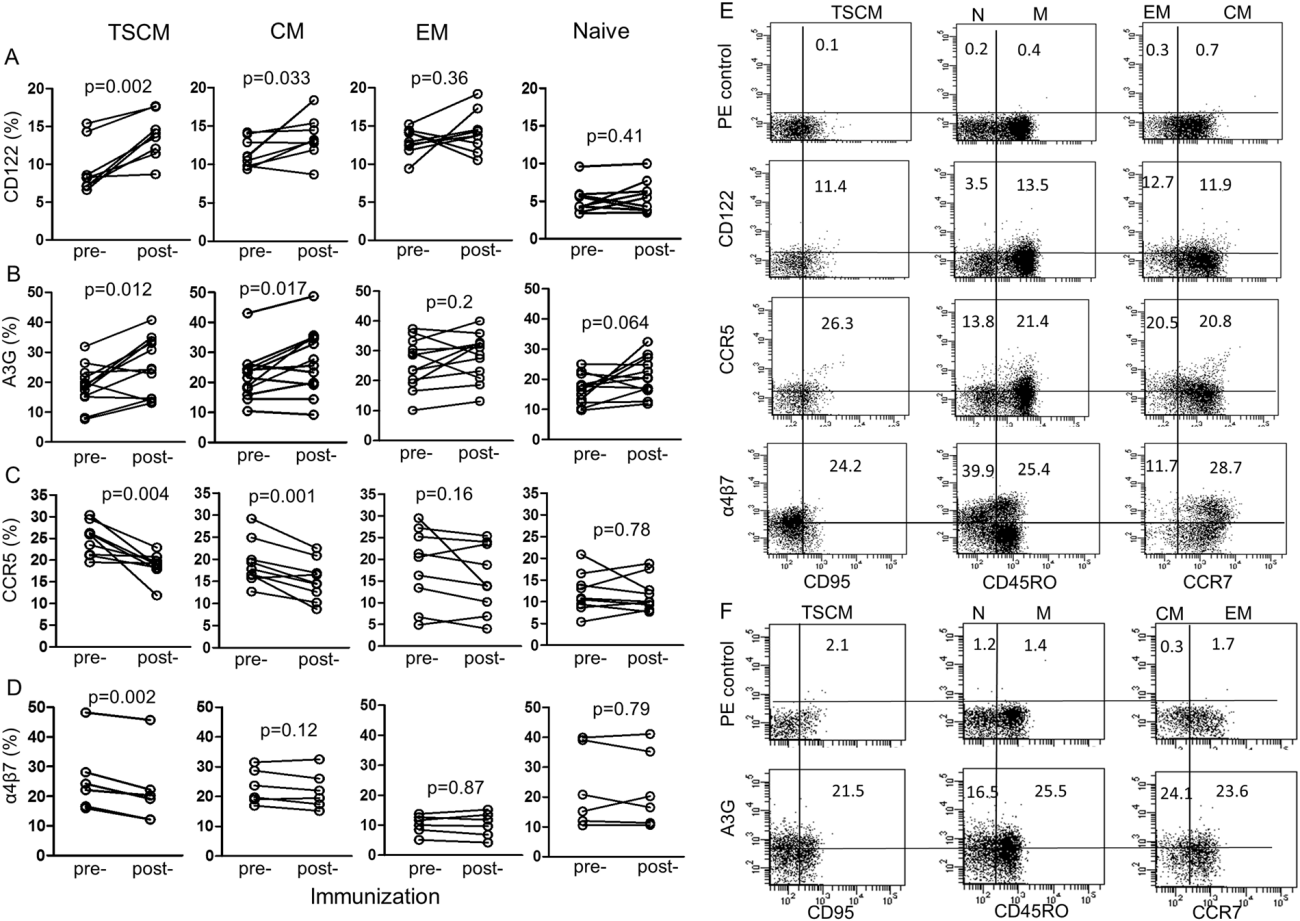
The effect of vaccination on the expression of (**A**) CD122, (**B**) A3G, (**C**) CCR5 and (**D**) intracellular α4β7 in CD4^+^ TSCM, central, effector memory and naive CD4^+^ T cells, before and 28 weeks after the last vaccination (n = 9, except (**B**) n = 12) from different subjects. However, the comparison between the different memory and naïve cells was with the same 9 samples). (**E**,**F**) Gating and identification of CD122, CCR5 α4β7 and A3G TSCM cells.

**Figure 3:**
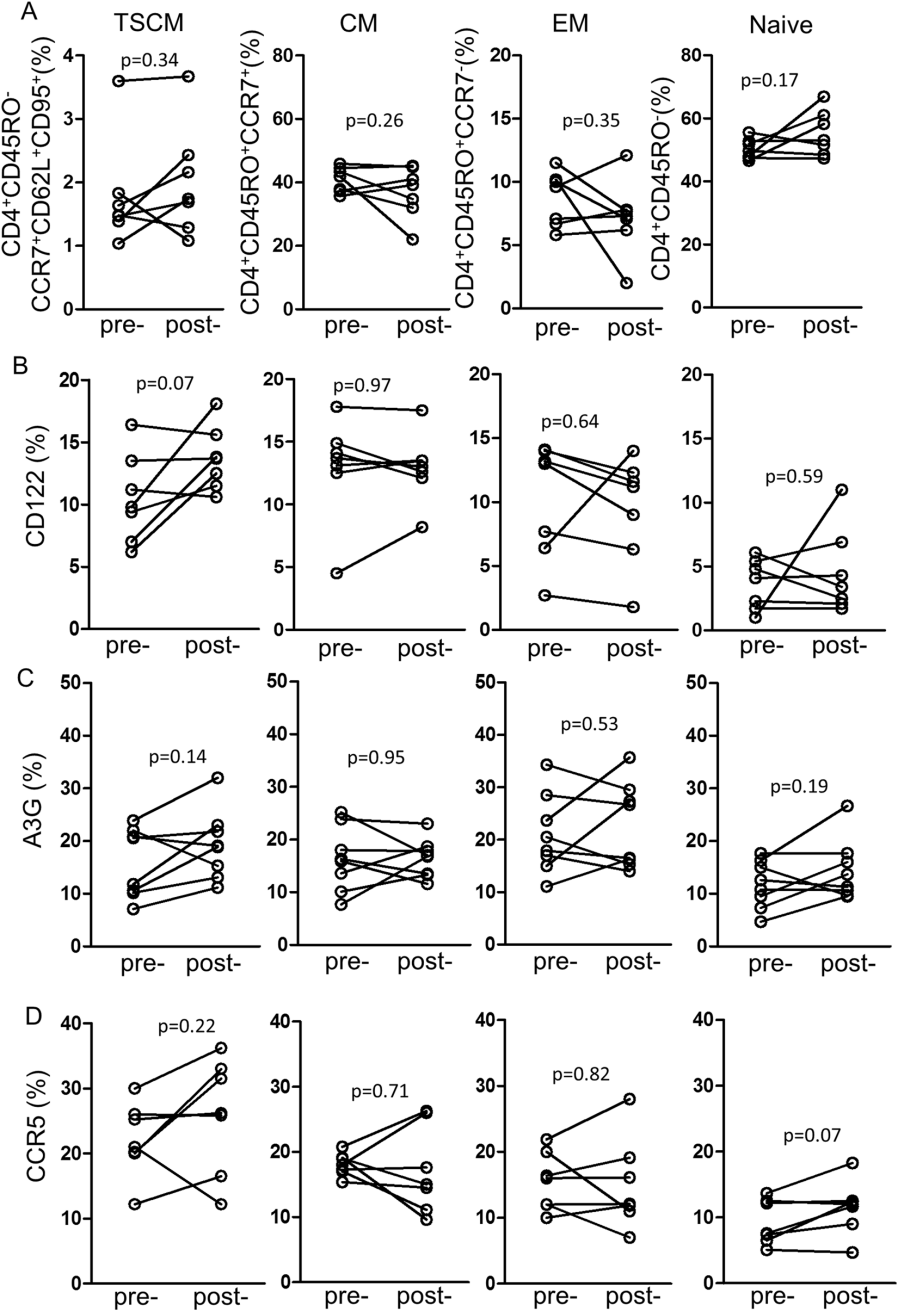
The effect of placebo on (**A**) TSCM, central, effector memory and naïve CD4^+^ T cells, (**B**) CD122, (**C**) A3G and (**D**) CCR5 in the corresponding CD4^+^ T cell subsets (n = 7) samples from different subjects but the comparison was as in Fig. 2. Gating and identification of these subsets of cells is similar to those illustrated in Fig. 2 (**E**,**F**).

The CD4^+^ TSCM were then studied to find out whether they express CD122, the β chain for IL-2 and IL-15 receptors, which might be activated by maIL-15 induced by the Canary pox, stress-mediated iHSP70^40, 41^ and IL-2 is produced by TSCM^10^. Significant increases in CD4^+^ CD122^+^ TSCM were found 28 weeks after vaccination (p = 0.002) and in CD45RO^+^ CCR7^+^ central memory T cells (p = 0.033), but not in CD45RO^+^CCR7^−^ effector memory or CD4^+^ CD45RO^−^ naive T cells (Fig. 2A). An illustration of flow cytometry is presented in Fig. 2E. The control placebo treated cohort showed no significant change in any of the 4 corresponding placebo control CD4^+^ T cells (Fig. 3A,B). Altogether, CD122 common receptors of IL-15 and IL-2 are likely to be elicited by an increase in maIL-15, through stress generated iHSP70, which activate and expand TSCM.

### Effect of the vaccine on the expression of APOBEC 3 G in TSCM

A3G is one of many restriction factors identified in TSCM^9^. We have studied the expression of A3G in CD4^+^ TSCM, before and after vaccination in relation to the persistence of memory and to T cell subsets^42^. A significant increase of A3G in CD4^+^ TSCM was found 28 weeks after vaccination (p = 0.012, Fig. 2B) and similarly to CD122 was significant in central (p = 0.017) but not effector memory (p = 0.20) or naïve CD4^+^ T cells. Flow cytometry illustration of A3G in TSCM and other T cell subsets are presented (Fig. 2F). Placebo controls failed to show significant changes (Fig. 3C).

### Effect of vaccination on the expression of CCR5 in CD4^+^ TSCM

In contrast to CD122 and A3G TSCM subsets, CD4^+^ TSCM expressing CCR5 were significantly decreased (p = 0.004, Fig. 2C) as were the central memory T cells (p < 0.001) but again not the effector or naïve CD4^+^ T cells. Flow cytometry is demonstrated in Fig. 2E. Corresponding studies were carried out with TSCM expressing CXCR4, which function as receptors for X4 HIV-1, but these failed to show any change (Supl. Fig. 1). The decrease in CCR5^+^ CD4^+^ T cells expressing coreceptors for R5 HIV-1, may contribute to the protective mechanism. It is noteworthy that the CD45RO^+^ CCR7^+^ central memory T cells followed a similar pattern to that of TSCM cells, with the CD122, A3G and CCR5^+^ TSCM, (Fig. 2A–C). The placebo controls failed to show significant changes (Fig. 3D).

### Effect of the vaccine on the expression of α4β7 integrin

The α4β7 integrin binds HIV-1 gp140 of CD4^+^ CCR5^+^ T cells, which home to the lamina propria of mucosal tissue of the gut and interacts with the addresin adhesion molecule on high endothelial venules. α4β7 was significantly decreased in CD4^+^ TSCM (p = 0.002), but not in the central or effector memory T cells (Fig. 2D,E), so its mucosal homing function may be diminished.

### Investigation of stress-induced HSP70 in the induction of CD4^+^ TSCM

**Figure 4:**
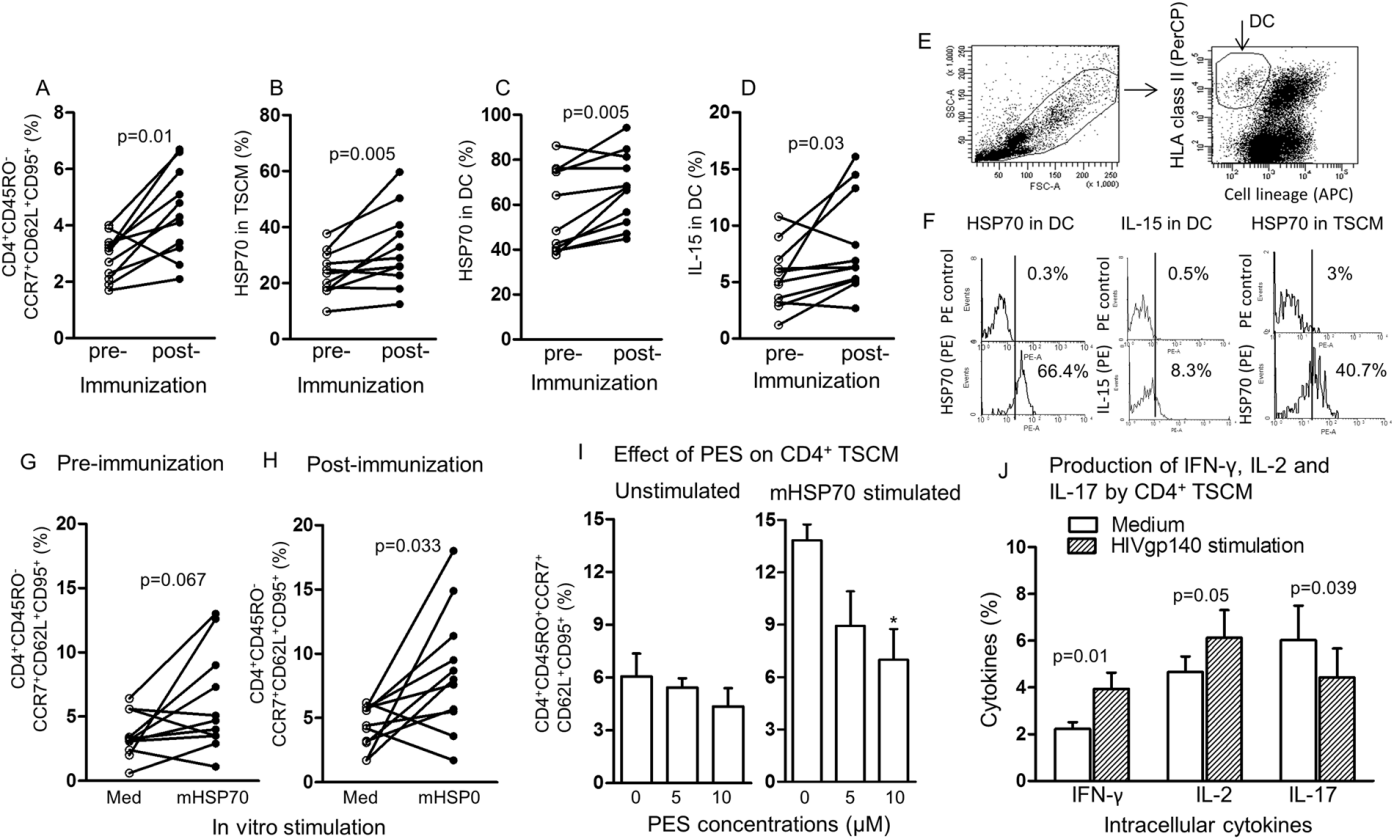
The effect of vaccination on inducible HSP70 in CD4^+^ TSCM and DC before and 28 weeks after immunization. (**A**) CD4^+^ TSCM, (**B**) HSP70 in CD4^+^ TSCM, (**C**) HSP70 in DC, (**D**) membrane associated (ma)IL-15 in DC. (**A–D**) Assays were performed on cells from the same vaccinees (n = 11). (**E**) Gating strategy used to identify DC. (**F**) Flow cytometry identifying HSP70 and IL-15 in DC, and HSP70 in TSCM. (**G**) The effect of stimulating CD4^+^ TSCM with microbial (m) HSP70 before and (**H**) after immunization. (**I**) The effect of dose dependent inhibition of HSP70 with PES in unstimulated and microbial (m)HSP70 stimulated CD4^+^ TSCM; *p value = 0.02, when compared with the untreated (0) µM of cells. (**J**) The effect of stimulating CD4^+^ TSCM with HIVgp140, compared with unstimulated cells on IFN-γ, IL-2 and IL-17 (n = 10 samples from different subjects).

To find out if the prime-boost vaccine of canary-pox (ALVAC-HIV-1, vCP1521) AIDSVAX B/E and Alum may have acted as stress agents we assayed iHSP70 in DC, which is a hallmark of cellular stress^43–45^. In a further cohort of 11 subjects we confirmed a significant increase in the post-immunization CD4^+^ TSCM (Fig. 4A, p = 0.01), upregulation of iHSP70 in TSCM or DC (Fig. 4B,C, p = 0.005) and IL-15 in DC (Fig. 4D, p = 0.03), but not in the placebos (data not shown). Microbial HSP70 was then used *in vitro* and confirmed that stress upregulates CD4^+^ TSCM, but significance was reached only in the post-immunised CD4^+^ TSCM samples (p = 0.033, Fig. 4G,H). Gating and the flow cytometry illustration of DC identification, IL-15 and iHSP70 expression in DC and iHSP70 in TSCM are presented in Fig. 4E,F. To confirm that iHSP70 is responsible for the increase in CD4^+^ TSCM, the cells were treated with PES (phenylethynesulfonamide), a small molecular inhibitor of iHSP70 function^46^. This showed a significant dose-dependent inhibition in post-immunised CD4^+^ TSCM, if stimulated *in vitro* with mHSP70 (Fig. 4I). These data are consistent with HSP70 induced cellular stress playing an important role in expanding and potentially self-replicating CD4^+^ TSCM.

### Characterisation of CD4^+^ TSCM by the production of cytokines

Further characterisation of the CD4^+^ TSCM by cytokines was pursued by intra-cellular staining. This showed that IFN-γ and IL-2 were significantly increased when stimulated with HIVgp140 of the post-immunized cells (p = 0.01 and p = 0.05, respectively Fig. 4J), whilst IL-17 was significantly decreased (p = 0.039). However, TNFα showed only a small increase on treatment with HIVgp140 (p = 0.2, Supl. Fig. 2A). As expected, the control anti-CD3 antibody stimulated, significantly greater cytokine response with IFN-γ, IL-2 and TNFα (p ≤ 0.001, Supl. Fig. 2A,B). The interpretation of these results is based on our demonstration that stress upregulates T bet and RORγt transcription factors, whereas FoxP3 is decreased, resulting in an overall enhanced Th1 polarisation^40^. This may enhance HIV-1 persistence during antiretroviral treatment^47^. However, the present human immunization results suggest that there is a divergence between Th1 (IFN-γ and IL-2) and Th17 (IL-17) cytokines and it is not clear which is the dominant partner.

### Pre- and post- immunization correlations between CD4^+^ TSCM and iHSP70, CD122, A3G, CCR5 and α4β7 subsets of TSCM

A major objective of this work was to find out which aspect of immune memory was not expressed optimally. To this end we have carried out pre- and post- immunization correlation analyses between CD4^+^ TSCM and its 4 subsets (Suppl. Fig. 3). These analyses failed to show significant correlations but only a direct trend of TSCM with HSP70 in DC from pre- (r = 0.37) to post-immunization (r = 0.54) and similarly with HSP70 in TSCM (0.45 to 0.56) respectively, consistent with stress-mediated HSP70 involvement in CD4^+^ TSCM generation. Interestingly, CD122 TSCM showed no correlation in the pre- or post- immunization cells (Suppl. Fig. 3C). However, A3G showed a reversal from an inverse trend of A3G before (r = −0.64, Suppl. Fig. 3D) to a slight direct trend after immunization (r = 0.02), which is a desirable direction of inhibition of HIV-1.

CCR5 subset of CD4^+^ TSCM cells showed conversion from a direct trend before (r = 0.19, Suppl. Fig. 3E) to an inverse trend (r = −0.46) after immunization with CD4 TSCM. Finally, α4β7 also converted, from a direct (r = 0.56, Suppl. Fig. 3F) to an inverse trend (r = −0.15), respectively. These results are consistent with immunization decreasing the proportion of CCR5^+^ CD4^+^ TSCM which inhibits their infectivity, whilst decrease in α4β7 TSCM inhibits homing function of CD4^+^ CCR5 T cells.

### Plasma CC chemokine upregulation and CD4^+^ T cell CCR5 co-receptor downmodulation

**Figure 5:**
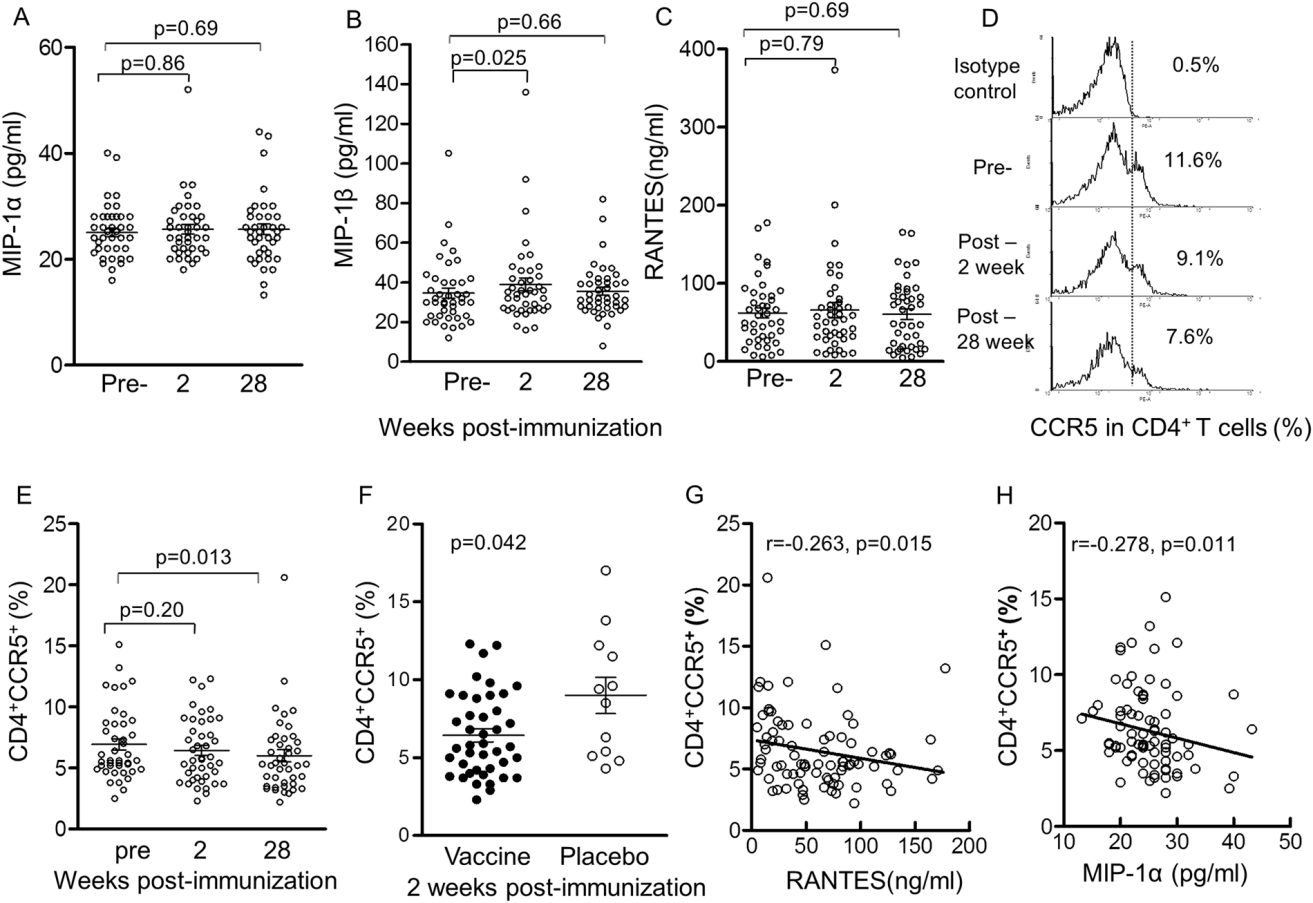
The effect of vaccination on production of CC chemokines and CCR5 in CD4^+^ T cells, 2 and 28 weeks after the last immunization. (**A**) MIP-1α in pg/ml, (**B**) MIP-1β in pg/ml, (**C**) RANTES in ng/ml, all from different subjects (**D**) Representative flow cytometry of (**E**) proportion of CCR5 in CD4^+^ T cells before and after immunization(%), (**F**) proportion of CCR5 in vaccinated, compared with the placebo group. (**G**) Inverse correlation between CD4 CCR5^+^ T cells and RANTES or (**H**) MIP-1α; Immunized (n = 42) and placebo (n = 12).

CC chemokines and A3G have been recognised to be important components of non-cognate or innate immunity. Hence, the 3 CC chemokines, CCR5 coreceptors of HIV-1 and the restriction factors A3G and Tetherin were studied in 42 blood samples from vaccinees and 12 placebos. Demonstrating induction of HSP70 suggested that the 3 CC chemokines may be upregulated^23–25, 48^ and these were studied in plasma by the Luminex bead assay, which showed significant increase in MIP-1β from 34.6 ± 2.59 pg/ml before to 39.1 ± 3.27 pg/ml, (p = 0.025) 2 weeks after the final immunization (Fig. 5B). MIP-1α and RANTES showed no significant change (Fig. 5A,C). In contrast CCR5 expression assayed by flow cytometry (Fig. 5D,E) was decreased from 6.92 ± 0.45 before to 6.43 ± 0.41 2 weeks and 5.99 ± 0.49 28 weeks after the final immunization, the latter reaching significance (p = 0.013, Fig. 5E). The vaccinees showed significantly lower CCR5 coreceptors than the placebo controls at week 2 after the final immunization (p = 0.042, Fig. 5F). Furthermore, significant inverse correlation was found between CCR5 and RANTES as well as MIP-1α at week 28 after the last immunization (p = 0.01, Fig. 5G,H). The results are consistent with the increase in CC chemokines early after vaccination, followed by downmodulation of CCR5.

### Upregulation of APOBEC3G (A3G) and Tetherin following immunization

**Figure 6:**
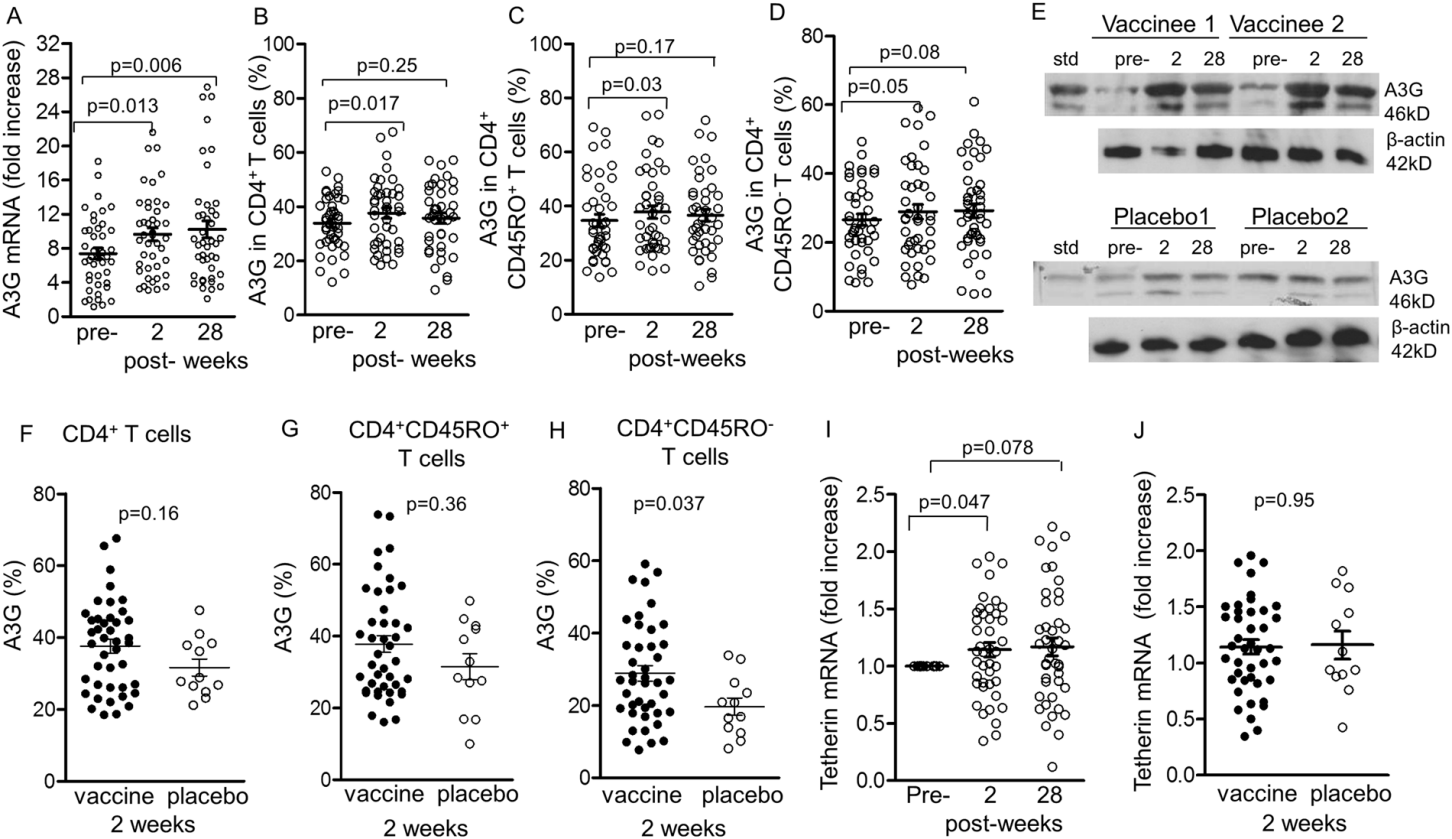
The restriction factors A3G and Tetherin 2 and 28 weeks after immunization. (**A**) A3G mRNA expression in PBMC examined by RT-PCR, (**B**) intracellular A3G in CD4^+^ T cells, (**C**) CD4^+^ CD45RO^+^ memory and (**D**) CD4^+^ CD45RO^−^ naïve T cells were all studied by flow cytometry. (**E**) Western blots of PBMC lysates from 2 representative vaccines and 2 placebo controls; the blots were trimmed above about 50 kD and below 40 kD. (**F**) A3G in vaccinees was compared with the placebo groups at 2 weeks in CD4^+^ (**G**), CD45RO^+^ (**H**) CD45RO^−^ T cells. (**I**) Tetherin mRNA was tested in PBMC before, 2 and 28 weeks after completion of immunization and (**J**) in vaccinees compared with placebos. Immunized (n = 42) placebo (n = 12, all from different subjects).

We have then studied the anti-viral restriction factors, A3G and tetherin, which are upregulated by iHSP70^27, 30^. A3G exerts intra-cellular anti-viral activity, so any HIV gaining entry into CD4^+^ T cells may then be inhibited. An important additional function of HSP70 is that it inhibits Vif- mediated ubiquitination and degradation of A3G, thereby retaining its function^30^. A3G mRNA assayed by RT-PCR showed significant fold-increase at week 2 (9.6 ± 0.7, p = 0.013) and at week 28 (10.2 ± 1.01, p = 0.006), compared with pre-immunization baseline level (7.4 ± 0.65, Fig. 6A, n = 42). A3G protein was then examined by flow cytometry following intracellular staining with anti-A3G antibodies. Significant upregulation of A3G protein expressing cells was found in CD4^+^ T cells (37.6 ± 1.9%, p = 0.017, Fig. 6B) and CD4^+^ CD45RO^+^ memory T cells (37.8 ± 2.3% p = 0.03, Fig. 6C) at week 2 after final immunization, compared with the pre-immunization samples (CD4^+^ T cells, 33.9 ± 1.5%). CD4^+^ CD45RO^−^ naïve T cells were also increased at week 2 (p = 0.05, Fig. 6D). Although higher levels of A3G protein were observed at week 28 in CD4^+^ T cells, CD4^+^ CD45RO^+^ memory and CD4^+^ CD45RO^−^ naive T cells, these failed to reach significant levels (Fig. 6B–D). The gating strategy is presented in Suppl. Fig. 4.

A3G protein production was further examined by Western blotting of PBMC lysates, which showed stronger bands 2 weeks after immunization and were maintained at least up to 28 weeks compared with the pre-immunization bands (Fig. 6E), representative blots from 2 immunized subjects). Two bands were seen, of which the upper was stronger than the lower, which is likely to be a breakdown product of the 44kDa upper band. Comparative Western blots in PBMC lysates from placebos showed only a slight increase in A3G protein at week 2 and 28, compared with the pre-immunization bands (Fig. 6E).

A comparison of A3G in PBMC from the vaccinated with those from placebo showed an increase in A3G protein in CD4^+^ and CD45RO^+^ memory and CD45RO^−^ naive T cells in the vaccinees (Fig. 6F–I) but significant levels were reached only in the CD4^+^ CD45RO^−^ naïve T cells (Fig. 6H). These results suggest that whilst the vaccinees showed significant increase in A3G compared with the pre-immunization samples, comparison with placebos also yielded higher levels in the vaccinees, though not all reached significance. As Alum was used in the controls for the 3rd and 4th treatment and Alum alone induces HSP70 as a stress response, which upregulates IL-15, IL-15Ra^45^ and in turn A3G^26^, this may account for the above findings in the placebo.

From previous studies we assumed that either specific HIV-1 antigens in the vaccine or HSP70 or both activated CD4^+^ T cells and induced A3G. To this end, we re-stimulated PBMC from the pre- and both post-immunization PBMC *in vitro* with 10 µg/ml HIV gp140, cultured for 3 days and intracellular A3G was assayed in the CD4^+^ T cell subsets. Re-stimulation increased significantly only CD4 CD45RO^+^ CCR7^−^ effector memory T cells at week 2 from 59.7 to 62.3% (p = 0.048, Supl. Fig. 5D), but not the CD4^+^ CD45RO^+^ or the CCR7^+^ central memory T cells (Supl. Fig. 5B,C). These results are consistent with the stress agent and not HIV antigen stimulating memory and central memory T cells.

We have then examined Tetherin, another HIV restriction factor by RT-PCR in the pre- and post- immunization blood samples. Tetherin mRNA showed a small but significant increase in the vaccinees, at 2 weeks after the last immunization, compared with the pre-immunization samples (p = 0.047, Fig. 6I), suggesting a vaccination specific effect. Surprisingly, there was no significant difference in Tetherin between the immunized and placebo groups (Fig. 6J), which is likely to be due to Alum used in the placebos.

**Figure 7:**
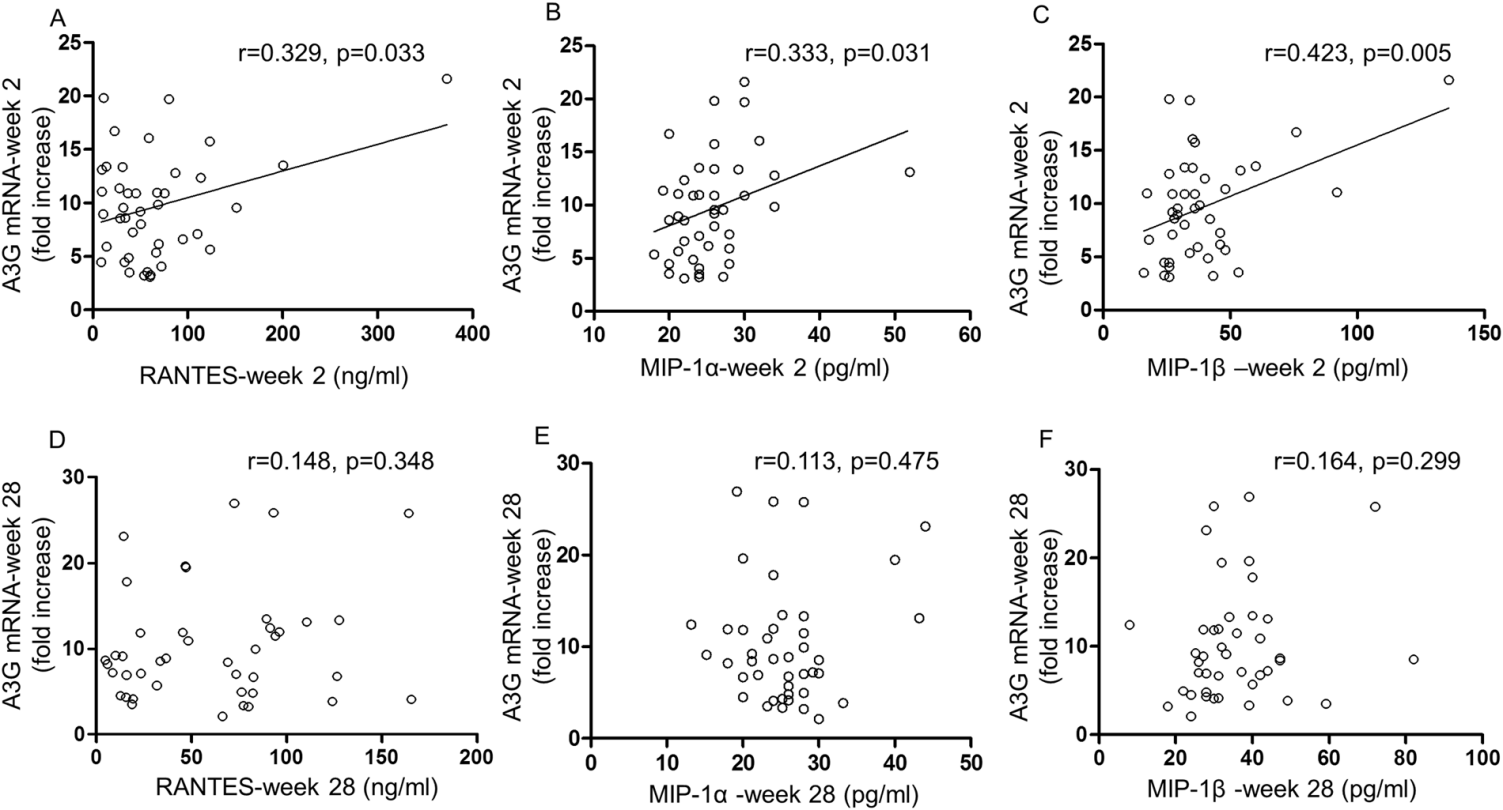
Correlation studies between RANTES, MIP-1α and MIP-1β CC chemokines and A3G mRNA showed significant direct correlation between them 2 weeks post-immunization (**A**,**B**,**C**), but was not maintained by 28 weeks (**D**–**F**); analysed by Spearman rank correlation coefficient; (n = 42 all from different subjects).

Interestingly, significant correlation was found between A3G mRNA, an innate intracellular restriction factor and RANTES (r = 0.329, p = 0.033), MIP-1α (r = 0.333, p = 0.031) and MIP-1β (r = 0.423, p = 0.005), extracellular innate factors downregulating CCR5. This was seen at week 2 (Fig. 7A–C), but not week 28 after immunization (Fig. 7D–F). These results are consistent with our earlier data that the 3 CC chemokines may induce upregulation of A3G by activating CCR5^26^ and this effect is induced early after immunization. Furthermore, they show correlation between two innate factors, which are involved in pre- and post-entry inhibition of HIV-1.

## Discussion

A robust immunological memory is critical for the function of any vaccine and has been inadequate in the RV144 vaccine. The efficacy of HIV-1 acquisition decreased from 60% in the 1^st^ year, to 36% in the 2^nd^ and 32.3% in the 3^rd^ year^1^, despite expressing significant Env-specific CD4^+^ CD45RO^+^ CCR7^−^ effector memory T cells^4^. This led us to examine TSCM cells reported in mice, NHP and humans^9, 10^, but this is the first report in vaccination. The proportion of CD4^+^, CD45RO^−^, CD62L^+^ CCR7^+^ CD95^+^ TSCM cells demonstrated by polychromatic markers was 2.33 ± 0.27% in the pre-immunized CD4^+^ T cells. Although it increased significantly after the last vaccination to 3.12 ± 0.36% (p = 0.018), this constituted a small proportion of self-replicating CD4^+^ T cells. An important finding was an increase in CD122 expressing CD4^+^ TSCM cells 28 weeks after the last immunization (p = 0.001). CD122 is a marker of the common IL-2 and IL-15 β chain receptor, the former being expressed by TSCM^9^ and the latter is induced in CD4^+^ T cells by iHSP70 following activation of ma IL-15 in DC^40, 45^. This is consistent with the paradigm that stress agents, to which the CD4^+^ TSCM cells are exposed, may regenerate their homeostatic pool by increasing maIL-15^40, 41^ and its receptor, thereby maintaining their prolonged function (over 10 years), as was demonstrated by genetically modified TSCM^49^.

CD4^+^ TSCM in HIV infection had been shown to harbour HIV and they play an important role in HIV persistence and latent HIV-1 reservoir^42, 50^. TSCM cells expressing CCR5 coreceptors of HIV-1 were significantly downregulated, thereby decreasing the infectivity of CD4^+^ TSCM. The R4 HIV-1 coreceptor CXCR4 expressing cells were not affected. Thus, 2 types of CD4^+^ TSCM cells were elicited by vaccination. CD122^+^ TSCM cells were upregulated, whilst CCR5^+^ TSCM were downregulated most likely by canarypox acting as a stress stimulating vector inducing HSP70. Homing of CCR5^+^ CD4^+^ T cells to mucosal tissues of the gut is mediated by α4β7 integrin. As immunization decreased the proportion of α4β7 and CCR5 subsets of TSCM, homing to mucosal tissues and availability of TSCM cells for infection will diminish. Indeed, TSCM are not found in mucosal tissues but are located in lymph nodes^10^. The HIV-1 restriction factor A3G subset of TSCM was significantly increased, which with the decreased expression of CCR5^+^ and α4β7^+^ subsets of TSCM will lead to greater inhibition of HIV-1 replication.

Vaccination in the RV144 clinical trial elicited iHSP70, which is the hallmark of cellular stress^43, 44^ and is likely to be involved in the mechanism of TSCM and innate immunity. HSP70 elicits CC chemokines, which downmodulate CCR5^22–25^, and increases A3G restriction factors^26^, as well as CD4^+^TSCM cells. Furthermore, HIV-1 virions carry a large number of host-derived antigens from previously infected cells, of which HSP70^51^ is expressed in high concentration, comparable to that of HIV pol protein. Thus, following exposure to HIV-1 infection the constitutive HSP70 in HIV virions may boost CD4^+^ TSCM, CC chemokines and A3G mediated inhibition of HIV-1 or SIV replication. This is consistent with the effect of iHSP70, exogenous mHSP70 or other stress agents inducing CD4^+^ TSCM. Treatment with PES, a small molecular inhibitor of iHSP70 function, inhibited CD4^+^ TSCM, suggesting that iHSP70 is involved in induction of CD4^+^ TSCM.

We suggest in addition to the established sequential CD4^+^ memory pathway I TSCM →CMC →EMC^9, 10^ a novel 2^nd^ innate immune pathway of CD4^+^ T cells (Supl. Fig. 6). The innate pathway consists of CD4^+^ TCSM generated subsets showing upregulation of IL2/IL-15 receptor expressing cells (CD122) and A3G TSCM cells, whereas the CCR5 and α4β7 subsets are downregulated. The overall trend of pre- and post-immunization between CD4^+^ TSCM and its subsets suggest that immunization favours HIV-1 inhibitory functions of TSCM, by increasing CD122 and A3G, whilst decreasing CCR5 and α4β7 expressing memory stem cells and the corresponding innate immunity.

In the RV144 samples significant increase in plasma MIP-1β within 2 weeks of vaccination was maintained 28 weeks after the last immunization. This was associated with downmodulation of CCR5 by 2 weeks, reaching significance by 28 weeks. A3G mRNA and its protein, as well as Tetherin and potentially other restriction factors, were significantly upregulated in CD4^+^ and CD45RO^+^ memory T cells by 2 weeks and was confirmed by Western blots. Importantly, each of the 3 CC chemokines showed significant direct correlation with A3G mRNA, suggesting that, they are likely to be stimulated by HSP70 activating CCR5 and/or TLR2 and TLR4^52, 53^. Restriction factors are dependent on type 1 IFN-stimulating genes and their expression in PBMC correlates with a decrease in viral load in IFN-treated patients^54^. Indeed, we have demonstrated that stress agents elicit large number of type 1 IFN induced genes^40^.

Stress induced CC chemokines reported here, elsewhere^4, 22–24^ and in the vaginal vaccination trial^25^ are consistent with an increase in MIP-1α and MIP-1β one day after IM administration of ALVAC in macaques or *in vitro* treatment with human PBMC^55^. A longitudinal study with the Canary pox vector showed that systemic vaccination significantly upregulates MIP-1α and MIP-1 β, in addition to IL-1β, TNF-α and MCP-1 early during the eclipse period of HIV-1 infection^55^. A significant correlation of the CC chemokines with the early development of A3G makes it likely that the innate restriction factor might also be increased in the eclipse period. Furthermore, cellular stress induces significant increase of cytokines, such as IFN-γ, IL-12, IFN-α, IL-1β, IL-6 and TGFβ^40, 45^, some of which were also demonstrated in the canary pox studies^55^, and IFN-γ with IL-2 in the present investigation. These data support the proposed stress-mediated effect early and possibly in the eclipse period of the RV144 vaccination trial.

Downregulation of CCR5 in CD4^+^ T cells has emerged as a major factor since the finding that subjects with homozygous ∆32 bp CCR5 deletion are protected from HIV-1 infection^11, 12^. Decrease of CCR5 expression in CD4^+^ T cells of humans and NHP had now been demonstrated following alloimmunization^14^, mucosal vaccination^25^, systemic vaccination (the present data), elite controllers or non-progressors in adults^18, 56, 57^, and in children^19^. This mechanism is also seen in natural infection with SIV of sooty mangabeys^17, 18^ and gene based methods, such as stem cell transplantation^58^. Many of these diverse strategies involve CD4^+^ TSCM.

Paradoxically CD4^+^ TSCM cells may also function as an HIV-1 reservoir, possibly because they express low concentrations of virus restriction factors^42^, promoting long-term virus persistence. The dual functional effect of long-term memory and HIV-1 latency of TSCM and the differential effect of the 4 subsets of CD4^+^ TSCM might be involved in the waning efficacy of the vaccine. We postulate that the timing in upregulation of CD122 and A3G expressing cells, associated with a decrease in CCR5 and α4β7 CD4^+^ TSCM cells may be critical in the outcome of the balance between HIV-1 infectivity linked latency of TSCM and potentially the beneficial long-term memory of TSCM^59^.

The overall analysis of cellular stress suggests the following mechanism.

1) Stress induces HSP70, which elicits NF-kB in DC and a series of phosphorylation signalling downstream in CD4^+^ memory T cells we have reported previously^40, 53^. 2) This induces CC chemokines, which downmodulates CCR5 co-receptors and inhibits pre-entry HIV-1. 3) Any HIV-1 virions, which escaped the pre-entry barrier may be inhibited post-entry by A3G and other restriction factors. 4) The adaptive immune phase, enhanced by innate immunity, plays a major role in inhibiting and eliminating any virus, which escaped the two early barriers by specific cytotoxic memory T cells, and B cell mediated antibodies^2–5^. The role of binding IgG antibodies, ADCC and the counteracting IgA antibodies are critical^2–5^. 5) Prolonged immune memory and innate responses may be mediated by a balance between increased CD122 and A3G, and decreased CCR5 and α4β7 expressing CD4^+^ TSCM pathways. We suggest that the dual stem cell memory interacting with the dual innate immunity may contribute to effective protection of HIV-1 infection and maintenance of prolonged immune memory.

## Methods

### Study Oversight

The protocol was approved by the ethics committees of the Ministry of Public Health, the Royal Thai Army, Mahidol University, and the Human Subjects Research Review Board of the U.S. Army Medical Research and Materiel Command. It was also independently reviewed and endorsed by the World Health Organization^1^. The ethical approval and relevant documents for the RV144 trial is in the public domain (registered at www.ClinicalTrials.gov, NCT00223080) and published in the first study report^1^. All volunteers were asked consent to participate in the trial and the Test of Understanding (TOU) was used to determine whether volunteers understood the trial and the pre-trial information regarding HIV, experimental vaccines, and their rights as a volunteer.

The present study is part of the RV144 phase III clinical trial, which was a community-based, randomized, multicentre, double-blind, placebo-controlled efficacy trial^1^. We obtained samples from 73 vaccinated subjects (38 male and 35 female) and 19 placebo controls (9 male, 10 female). Thai men and women were between 18 and 30 years of age and they were not infected with HIV before or after the trial. They were recruited from the community without regard to HIV risk (i.e., community risk). Women were counselled to practice effective contraception until 3 months after the last vaccination; pregnant and breast-feeding women were excluded^1^. For the TSCM studies we used 35 vaccinated and 7 placebo controls and for the innate immunity studies we used 42 vaccinated subjects and 12 placebo controls. In both cohorts there were similar number of male and female subjects.

### PBMC and plasma samples

Blood was withdrawn by venepuncture, PBMC and plasma were prepared using 8 ml sodium citrate Vacutainer® Cell preparation tubes (CPT™). Separated PBMC were cryopreserved in RPMI medium and plasma stored at −80 °C^1^. Vaccine and the immunization procedure was published previously^1^. Samples collected before start of immunization, and 2 and 28 weeks after completion of immunization (week 24) were used to analyse the concentrations of three CC chemokines, TSCM, expression of cell surface CCR5 and intracellular APOBEC3G, Tetherin and HSP70. All the data presented are from different immunized or control subjects and all the comparative assays of TSCM – CM – EM - and naïve cells are from the same subjects.

### Assay of CD4^+^ and CD8^+^ TSCM and inhibition of the CD4^+^TSCM cells by PES

The pre-immunized and post-immunized samples were then analysed in parallel in each assay. The viability of thawed cells was checked by trypan blue exclusion and was greater than 95%. Phenotypic expression of CD4 and CD8 memory TSCM cells were identified by polychromatic flow cytometry, using antibodies to CD45RO, central memory cells by expressing CD4^+^ CD45RO^+^ CCR7^+^ and effector memory cells by CD4^+^CD45^+^CCR7^−^ cells^39^. CD4 or CD8 TSCM cells were identified in PBMC by antibodies to CD45RO (PE-Cy7), CD62L (APC-Cy-7), CCR7 (PerCP) and CD95 (FITC). All conjugated antibodies were purchased from Biolegend UK. In addition, antibodies to CD122, CCR5, CXCR4 (PE-conjugated, all from Biolegend) and α4β7 (clone Act-1, obtained from NIBSC, Potters Bar, UK) were used to define TSCM subsets. 5 × 10^5^ PBMC were incubated with a combination of fluorochrome conjugated antibodies (Biolegend UK) for 30 min at 4 °C, washed and then analysed by the FACScanto II flow cytometer, using DIVA software. A3G expression in TSCM was then assayed by intra-cellular staining as described below.

To determine the specificity of HSP70 in stimulating TSCM, dose dependent inhibition was carried out with the HSP70 inhibitor, 2-phenylethynesulfonamide (PES, Calbiochem, Merck, UK). PBMC were stimulated with mHSP70 (20 µg/ml) in the presence of various concentrations (5–100 µM) of PES. After 5 day culture, TSCM were analysed by flow cytometry.

### Assay of inducible HSP70 (iHsp70) in DC

DC were analysed by flow cytometry and defined as live, lineage negative, HLA DR^high^ cells. PBMC were surface stained with lineage cocktail antibodies (conjugated with APC), against CD3, CD14, CD16, CD19, CD20, CD56 and HLA-DR PE Cy7 (clone L243), both from Biolegend (cat 348803) and fixable viability dye e780 (eBioscience). Cells were then fixed, permeabilised with the intracellular fixation and permeabilization buffers (eBioscience) and then stained with iHSP70 PE (clone C92F3A Stressgen) or an isotype control antibody.

### Assay of TSCM cytokines by intracellular staining

CD4^+^ TSCM produced cytokines, IFN-γ, IL-2, IL-17 and TNFα were detected by intracellular staining the cells with PE-conjugated antibodies (Biolegend) following restimulation of cells with PMA, after 4 days *in vitro* culture of cells in the presence 10 µg/ml of HIVgp140 antigen (NIBSC, Potters Bar, UK).

### Assay of RANTES, MIP-1α, and MIP-1β in plasma

Quantitation of RANTES, macrophage inflammatory protein 1α (MIP-1α), and MIP-1β (CCL-5, CCL-3, and CCL-4, respectively) was carried out by a Luminex bead assay using Fluorokine multianalyte profiling (MAP) kits (R&D, Oxford, United Kingdom), as described previously^25^.

### Flow cytometry analysis of cell surface expression of CCR5 on CD4^+^ T cells

CCR5 expression on CD4^+^ T cells was identified by incubating 1 × 10^6^ PBMCs with antibodies specific to CCR5 (BD Biosciences, United Kingdom). After 20 min the cells were washed and analysed by flow cytometry, and live cells were gated and expressed as the proportion of CCR5 on CD4^+^ T cells.

### Preparation of RNA and cDNA

PBMC (2 × 10^6^) were thawed from cryo-preserved samples into RPMI 1640 medium supplemented with 10% FCS. After centrifugation at 500 g for 5 min, the cell pellets were washed with PBS. RNA was isolated using a Total RNA Isolation Kit (Promega, UK), quantified using the spectrophotometer (GeneQuant II, Pharmacia Biotech), and cDNA was generated from RNA by using the Reverse Transcription System (Promega), according to the manufacturer’s instructions. Cryo-preserved PBMC isolated from a healthy donor (National Blood Service) was thawed and RNA was prepared as the internal control.

### Real-time PCR for A3G and tetherin mRNA

Relative amount of A3G mRNA was quantified by real-time PCR (ABI Prism 5700) using the PlatinumSYBR green qPCR SuperMix-UDG without ROX (Invitrogen Life Technologies). The primers for A3G and GAPDH were described elsewhere^26^. When assaying the samples for A3G and GAPDH, an internal control was included in each run under the same conditions and the relative amount of A3G in the samples was determined by comparing with the Ct values of the internal control sample. The results were expressed as the fold increase of internal control. Tetherin/bst2 mRNA levels were measured using an ABI primer/probe qRT-PCR set and calculated relative to GAPDH mRNA on an ABI 6700 machine.

### Investigations of A3G protein by flow cytometry

Intracellular A3G protein expression on CD4^+^ T cells was assayed by intracellular staining with anti-A3G MAbs, as described elsewhere^35^. MAbs to A3G and isotype control antibody from ABDserotec were conjugated with fluorescein isothiocyanate (FITC) using a LYNX rapid fluorescein antibody conjugation kit (ABD Serotec, Oxford, United Kingdom). Optimal concentration was determined by serial dilutions and the reproducibility was tested by staining the thawed PBMC from the same subjects at different times; variations were less than 5%.

Human CD4^+^ memory and naïve T cell subsets were identified using antibodies to CD45RO as described above. After cell surface staining the cells (2 × 10^5^) were washed and fixed with a fixation buffer for 3 min (eBioscience, Insight Biotechnology, London, United Kingdom), followed by treatment with the permeabilization buffer (eBioscience). FITC-conjugated A3G antibody (10 u l at 10 μg/ml) was added to the cell pellets, and following 30 min of incubation, the cells were washed and analyzed by flow cytometry on a FACSCanto II flow cytometer (BD Biosciences) using FACSDiva software. To assay the effect of specific antigen re-stimulation on A3G production, aliquots of 200 µl PBMC (3 × 10^6^/ml) were incubated in RPMI medium supplemented with 10% FCS, 2 mM glutamine and 100 µg/ml of penicillin and streptomycin, in the presence or absence of 10 µg/ml of HIV-1_MN_ gp120. After 3 day culture, cells were assayed for A3G expression by flow cytometry.

### Statistical analysis

All results are expressed as mean (±sem). The non-parametric paired t test (Wilcoxon signed rank test) was used for analysis of significance between pre- and post-immunized samples. Mann Whitney test was used to analyse the significance between vaccine and placebo groups. Spearman rank or Pearson correlation coefficient was applied for analyses of correlations. Probability value (p) < 0.05 was considered to be significant.

## Electronic supplementary material

Legend to Supplementary Figures
