# Supplementary material for: A novel mechanism linking memory stem cells with innate immunity in protection against HIV-1 infection
Source: Sci Rep. 2017 Apr 21;7:1057. doi: 10.1038/s41598-017-01188-3 (PMC5430909; doi:10.1038/s41598-017-01188-3)

**A novel mechanism linking memory stem cells with innate immunity in protection against HIV-1 infection**

**Running Title:** Stem cell memory and innate immunity to HIV-1

Yufei Wang^1^, Trevor Whittall^1^, Stuart Neil^2^, Gary Britton^1^, Mukesh Mistry^1^, Supachai Rerks-Ngarm^3^, Punnee Pitisuttithum^4^, Jaranit Kaewkungwal^4^, Sorachai Nitayaphan^5^, Xuesong Yu^6^, Alicia Sato^6^, Robert J. O'Connell^7^ Nelson L. Michael^7^, Merlin L. Robb^7,8^, Jerome H. Kim^7,9^ and Thomas Lehner^1*^.

^1^ Mucosal Immunology Unit, ^2^Dept. of virology; Kings College London, ^3^Department of Disease Control, Ministry of Public Health, Nonthaburi, ^4^Faculty of Tropical Medicine, Mahidol University, Bangkok, ^5^Armed Forces Research Institute of Medical Sciences, Bangkok, Thailand, ^6^Statistical Center for HIV/AIDS Research and Prevention, Fred Hutchinson Cancer Research Center Seattle, ^7^US Military Research Program, Walter Reed Army Institute of Research, Silver Spring, ^8^Henry Jackson Foundation for the Advancement of Military Medicine, Bethesda, MD,^9^ International Vaccine Institute Seoul, Korea.

**Corresponding author**: *Thomas Lehner (Thomas.lehner@kcl.ac.uk)

**Legend to Supplementary Figures**

Supl. Fig.1 The effect of vaccination on CXCR4 coreceptors in (A) CD4^+^ TSCM, (B) CD4^+^ central and (C) effector memory, and (D) naïve cells. n=9 from different subjects but the comparison of 4 subsets of cells were carried out on the same 9 samples.

Supl. Fig.2 The effect of anti-CD3 antibody stimulation of PBMC from post-immunized subjects on (A) TNFα and compared with that of HIV gp140, (B) IFN-γ, IL-2 and IL-17 cytokines.(n=10 different subjects and cytokine assays carried out on the same 10 samples.

Supl. Fig.3 Pre- and post-immunization correlation trends between CD4+ TSCM and HSP70 in DC (A), in TSCM (B), and CD122 (C), A3G (D), CCR5 (E), and α4β7 (F) (n=18-22 per group). The thin lines show direct and inverse trends pre- and post-immunization and the correlation r values, which failed to reach significance. ○ pre-immunization, ▪ post-immunization.

Supl. Fig. 4 Gating strategy of A3G in CD4 CD45RO^+^ memory T cells; the data are presented in Fig. 6.

Supl. Fig.5 Effect of in vitro antigen (HIVgp140) re-stimulation on A3G production in (A) CD4^+^, (B) CD45RO^+^ memory , (C)CD45RO^+^CCR7^+^ central and (D) CDD45RO^+^CCR7^-^ effector memory T cells. ○ medium.▪ HIVgp140. (n=42 different subjects per group).

Supl. Fig. 6 Diagrammatic representation of the 2 pathways generated by CD4^+^ memory stem cells (TSCM); I Memory pathway, II Innate pathway of the 4 subsets of CD4+ TSCM; IL-15/IL-2 receptors, APOBEC3G restriction factors, CCR5 coreceptors and α4β7 integrins. ↑ increase, ↓ decrease.


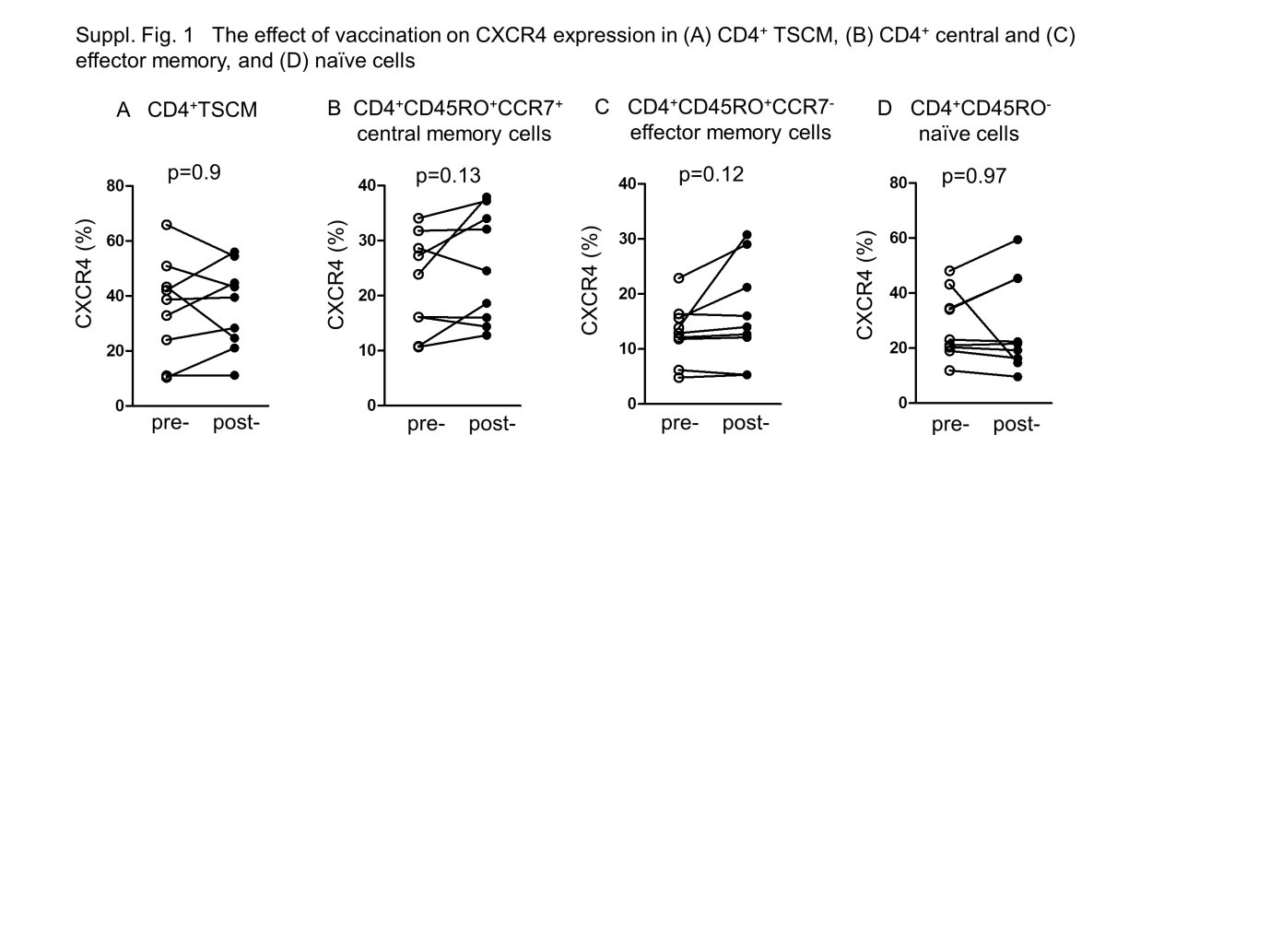


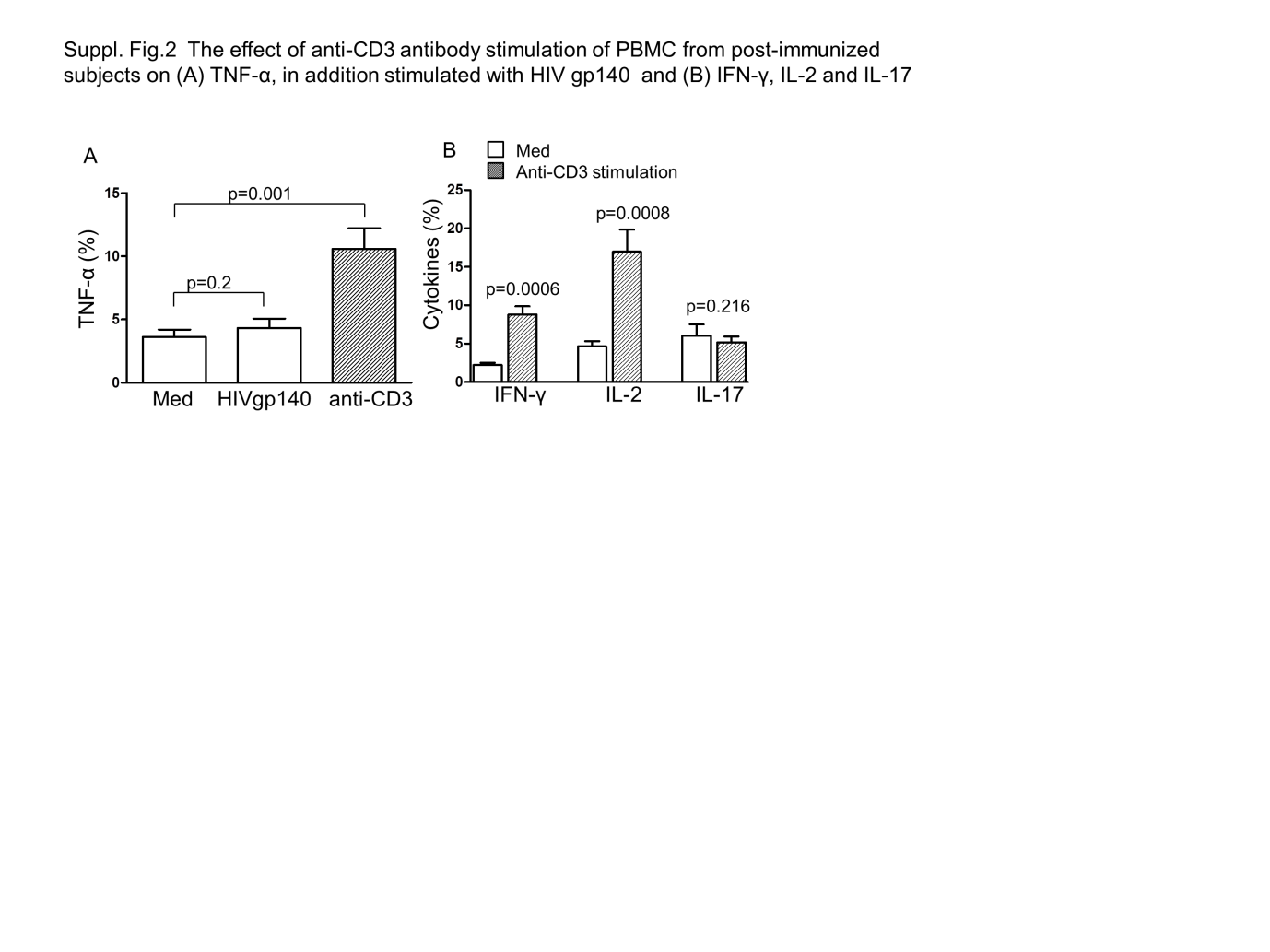

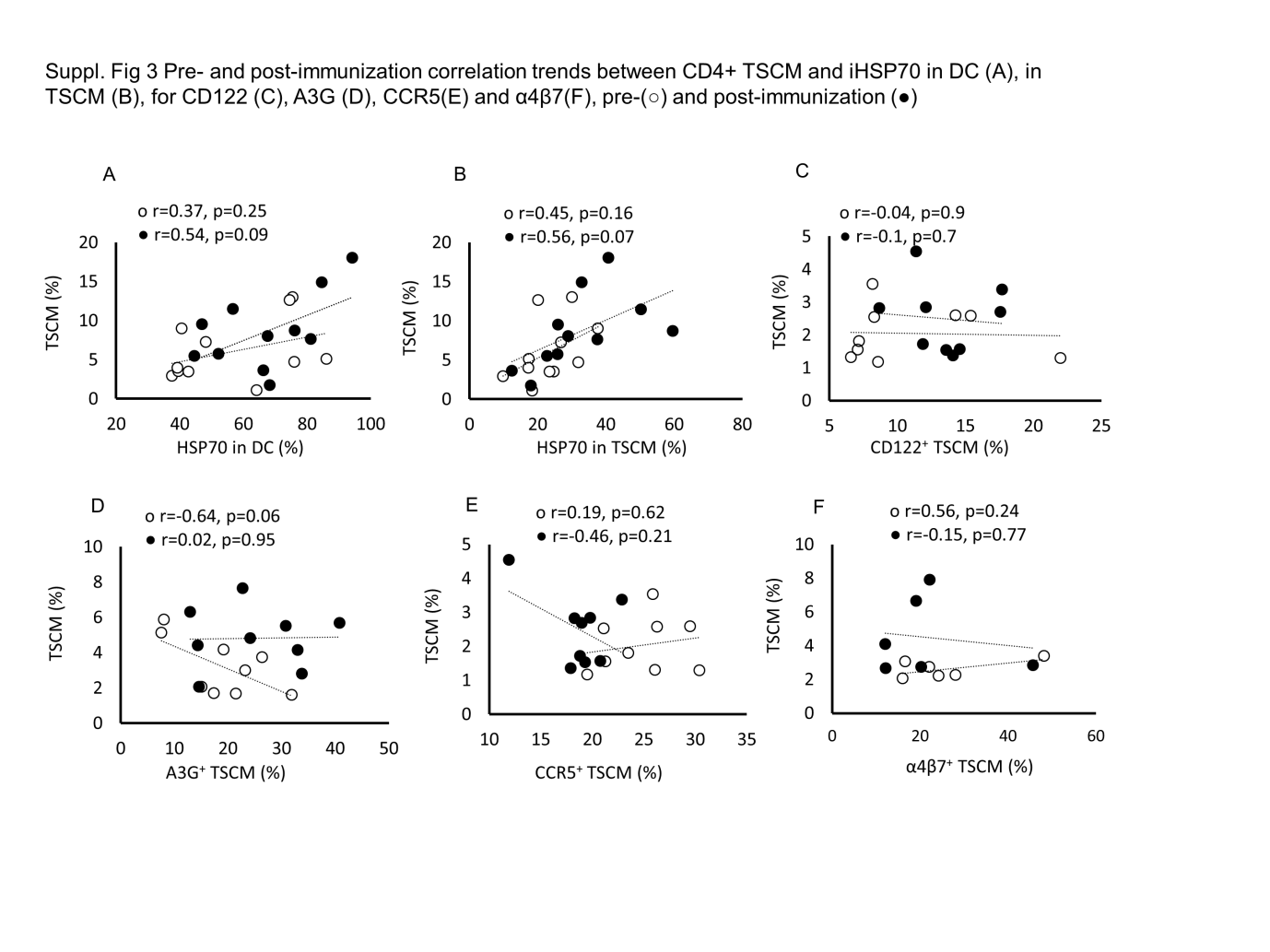


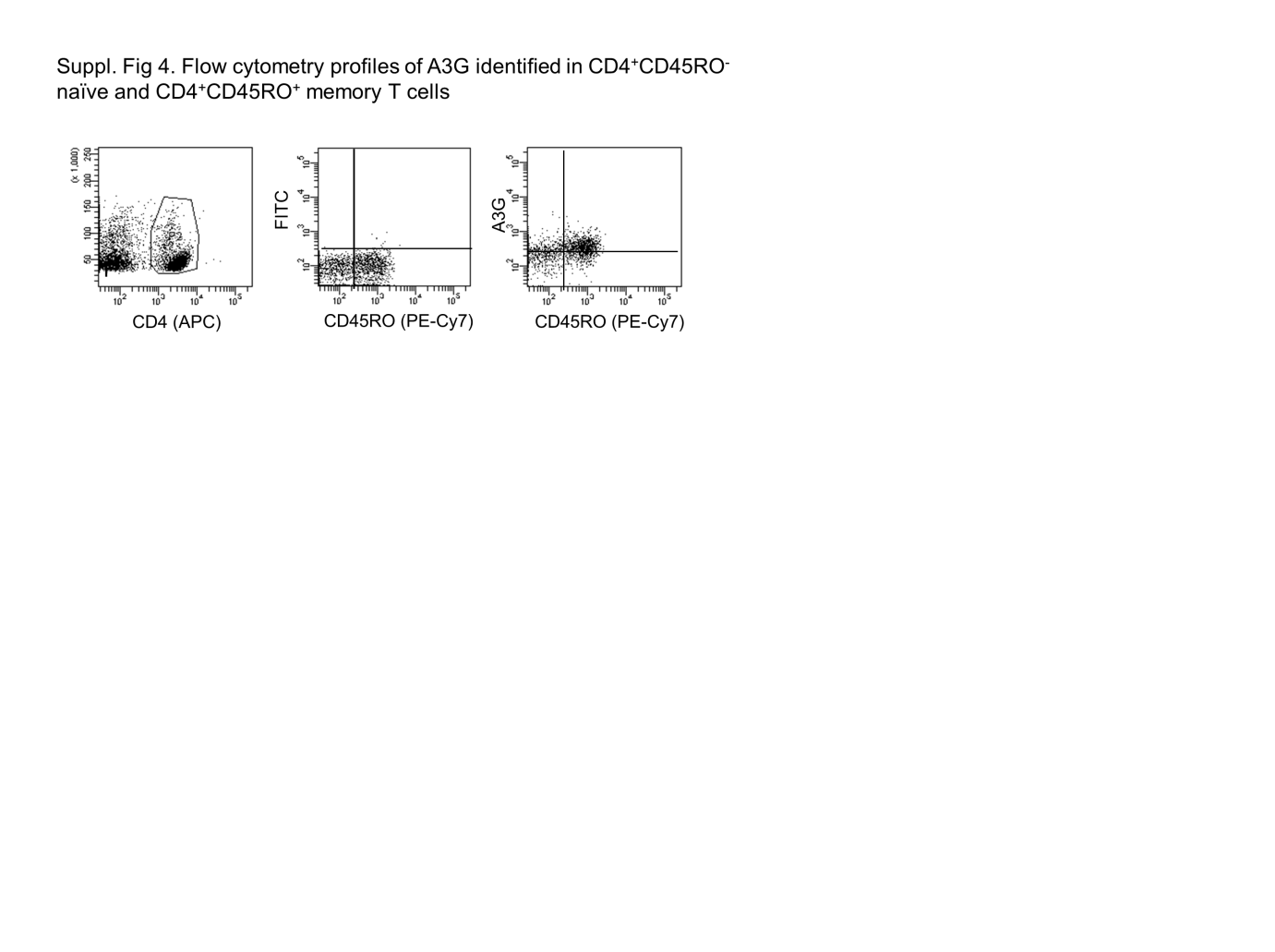


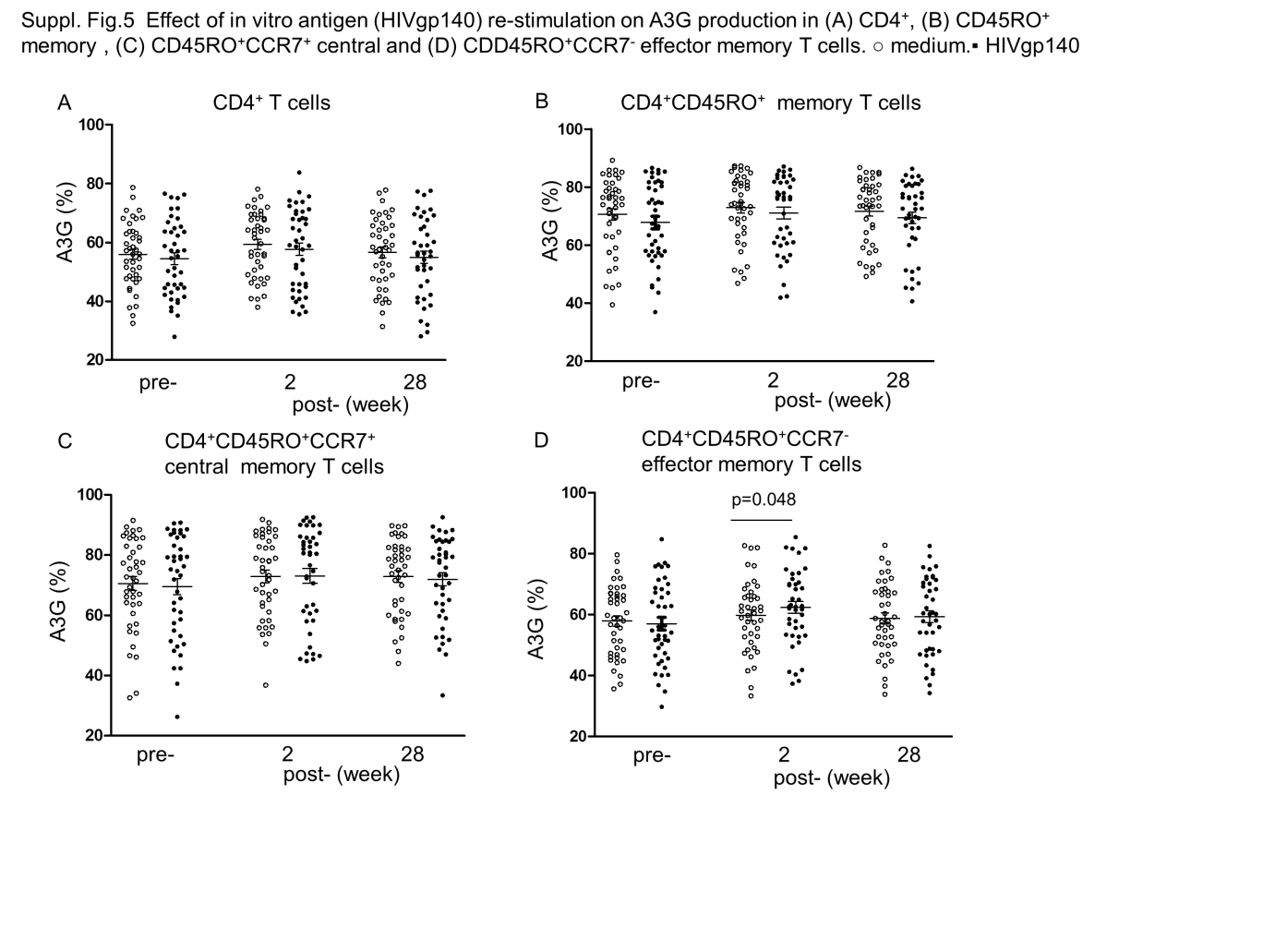


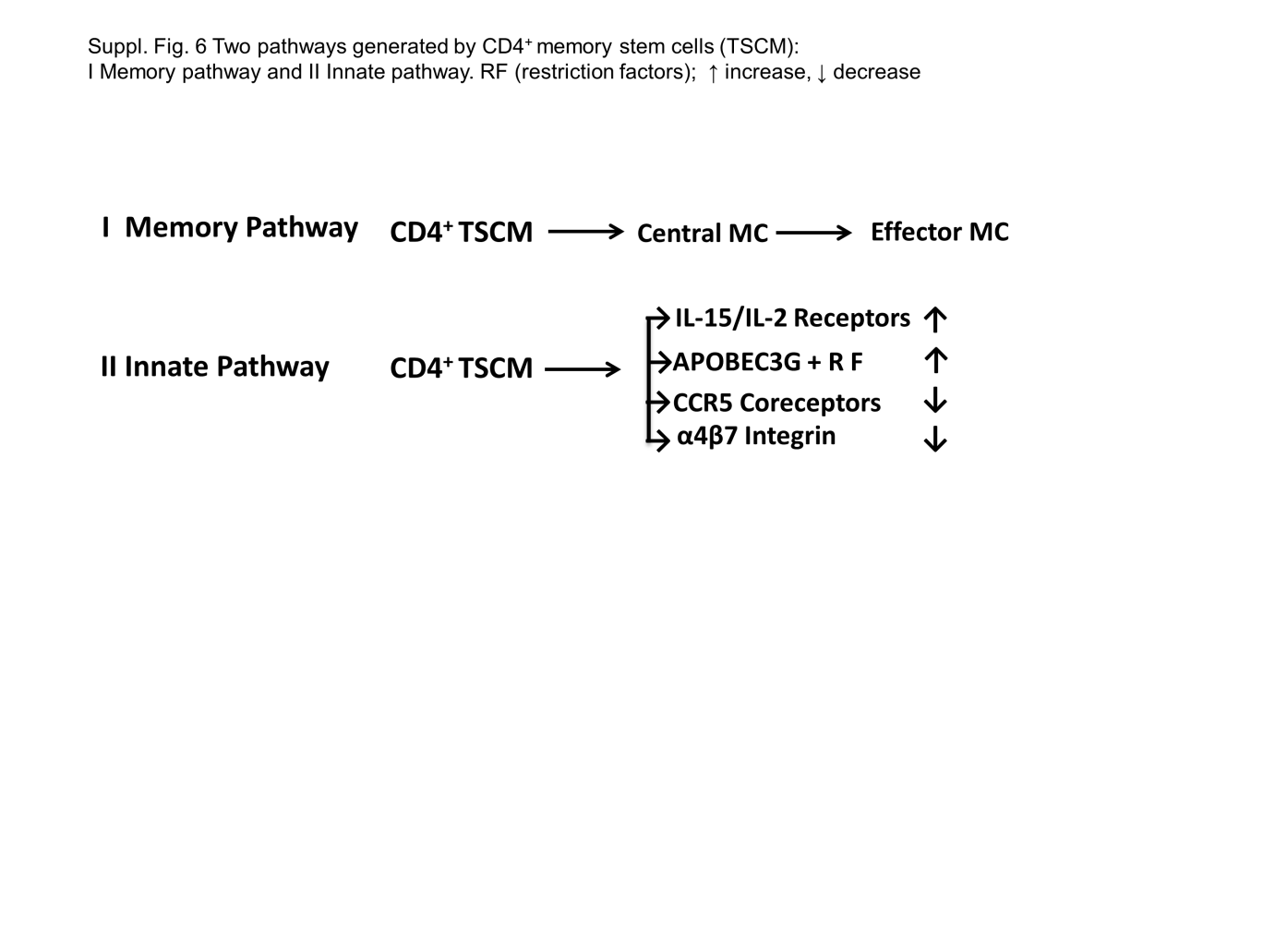

Supplement: Supplementary file 1 — Legend to Supplementary Figures [file 41598_2017_1188_MOESM1_ESM.doc]
